# Impact of pharmacy-led medication reconciliation on admission to internal medicine service: experience in two tertiary care teaching hospitals

**DOI:** 10.1186/s12913-019-4323-7

**Published:** 2019-07-16

**Authors:** Lamis R. Karaoui, Nibal Chamoun, Jessica Fakhir, Wael Abi Ghanem, Sarah Droubi, Abdul Rahman Diab Marzouk, Nabila Droubi, Hiba Masri, Elsy Ramia

**Affiliations:** 1 Department of Pharmacy Practice, School of Pharmacy, Lebanese American University, P.O. Box S-23, Byblos, Lebanon; 2 Saint George Hospital – University Medical Center, Pharmacy Department, Beirut, Lebanon; 3 Makassed General Hospital, Pharmacy Department, Beirut, Lebanon

**Keywords:** Medication reconciliation, Hospital admission, Drug safety, Patient safety, Transition of care, Quality improvement, Hospital pharmacy, Lebanon

## Abstract

**Background:**

The Institute for Healthcare Improvement identifies medication reconciliation as the shared responsibility of nurses, pharmacists, and physicians, where each has a defined role. The study aims to assess the clinical impact of pharmacy-led medication reconciliation performed on day one of hospital admission to the internal medicine service.

**Methods:**

This is a pilot prospective study conducted at two tertiary care teaching hospitals in Lebanon. Student pharmacists who were properly trained and closely supervised, collected the medication history, and pharmacists at the corresponding sites performed the reconciliation process. Interventions related to the unintended discrepancies were relayed to the medical team. The main outcome was the number of unintended discrepancies identified. The time needed for medication history, and the information sources used to complete the Best Possible Medication History were also assessed. The unintended discrepancies were classified by medication class and route of medication administration, by potential severity, and by proximal cause leading to the discrepancy. For the bivariate and multivariable analysis, the dependent variable was the incidence of unintended discrepancies. The “total number of unintended discrepancies” was dichotomized into yes (≥ 1 unintended discrepancy) or no (0 unintended discrepancies). Independent variables tested for their association with the dependent variable consisted of the following: gender, age, creatinine clearance, number of home medications, allergies, previous adverse drug reactions, and number of information sources used to obtain the BPMH. Results were assumed to be significant when p was < 0.05.

**Results:**

During the study period, 204 patients were included, and 195 unintended discrepancies were identified. The most common discrepancies consisted of medication omission (71.8%), and the most common agents involved were dietary supplements (27.7%). Around 36% of the unintended discrepancies were judged as clinically significant, and only 1% were judged as serious. The most common interventions included the addition of a medication (71.8%) and the adjustment of a dose (12.8%). The number of home medications was significantly associated with the occurrence of unintended discrepancies (ORa = 1.11 (1.03–1.19) *p* = 0.007).

**Conclusions:**

Pharmacy-led medication reconciliation upon admission, along with student pharmacist involvement and physician communication can reduce unintended discrepancies and improve medication safety and patient outcomes.

**Electronic supplementary material:**

The online version of this article (10.1186/s12913-019-4323-7) contains supplementary material, which is available to authorized users.

## Background

Medication errors remain a significant patient safety concern and a financial burden in hospitalized patients in the United States and Europe [1–3].At transitions of care, more than 50% of medication errors occur, and up to 67% of medication histories contain one or more errors [4, 5]. With the rising number of patients receiving multiple medications and the complex medication therapy management, medication reconciliation becomes warranted. In many organizations, the implementation of medication reconciliation at all transitions of care (admission, transfer, and discharge) helped prevent Adverse Drug Events (ADEs) [6] and reduce medication errors [7]. In a recent randomized controlled study conducted in Oman, the implementation of medication reconciliation on admission and discharge reduced rates of preventable adverse drug events from 16 to 9.1%, *p* = 0.009 [8]. In another recent prospective interventional study conducted to determine the effect of medication reconciliation in two ICU settings in the Netherlands, the proportion of patients with ≥1 medication transfer error at ICU admission was reduced from 45.1 to 14.6% (OR_adj_ 0.18 [95% CI 0.11–0.30]) and after ICU discharge from 73.9 to 41.2% (OR_adj_ 0.24 [95% CI 0.15–0.37]) [9].

Effective January 2018, the Joint Commission named maintaining and communicating accurate patient medication information as a National Patient Safety Goal for hospitals [10].

The process of medication reconciliation performed by healthcare professionals (HCPs) has been previously defined [11, 12]. During this process, HCPs obtain the best possible medication history (BPMH), from patients and their families in order to identify and resolve any unintended discrepancies [10–12]. To date, there is no consensus with regards to who should have ownership of the process and who should be assigned to complete the medication list [13–15]. Other challenges include the absence of a standardized process, time limitation, and patients’ or physicians’ lack of familiarity with some medications [15]. The Institute for Healthcare Improvement identifies reconciliation as the shared responsibility of nurses, pharmacists, and physicians, where each has a defined role [15].

The American Society of Health-System Pharmacists advocates for the pharmacist’s role in medication reconciliation [14]. In recent studies, the pharmacist’s involvement in medication reconciliation at admission and discharge was shown to decrease the number of medication errors, improve patient safety, and reduce costs associated with health resource utilization [16–24]. In a meta-analysis that compared the effectiveness of pharmacist-led medication reconciliation interventions to usual care, the pooled analysis from 17 studies involving 21,342 adult patients showed a substantial reduction of 67, 28 and 19% in adverse drug event-related hospital revisits (RR 0.33; 95% CI 0.20 to 0.53), emergency department (ED) visits (RR 0.72; 95% CI 0.57 to 0.92) and hospital readmissions (RR 0.81; 95% CI 0.70 to 0.95) in the intervention group as compared to the usual care group, respectively [24]. Furthermore, studies proved that pharmacy students and technicians are accurate and efficient when included in the medication reconciliation process; they decrease costs and provide support to other HCPs [25].

In Lebanon, a well-defined practice for medication reconciliation does not exist. The implementation of medication reconciliation in Lebanese hospitals does not keep pace with international standards. Nurses or physicians are currently involved in taking the medication history, while the role of the pharmacist is underestimated. A recent survey evaluating general hospital pharmacy practice in Lebanon revealed that around 41% of hospitals perform medication reconciliation upon admission, transfer of care, and discharge. Teaching hospitals were more likely to perform medication reconciliation than non-teaching hospitals; and pharmacy services in teaching hospitals seemed to be more advanced cooperating with affiliated medical schools [26]. A previous study, not directly focusing on medication reconciliation, showed that student pharmacists and faculty identified more than 1000 medication-related problems, 4.3% of which were related to medication reconciliation [27]. A study assessing hospital pharmacy medication reconciliation practice in Jordan (a neighboring country) showed relatively low awareness of the concept and policy of medication reconciliation process among Jordanian pharmacists [28]. To our knowledge, there are no published studies addressing medication reconciliation in Lebanon.

The primary objective of this study was to assess the clinical impact of pharmacy-led medication reconciliation performed on adults patients admitted to the Internal Medicine (IM) services with ≥1 chronic medication, on day one of hospital admission, measured by the incidence of unintended medication discrepancies identified. The secondary objectives were to assess the time needed for medication history, and the information sources used to complete the BPMH. Other secondary objectives were to classify the unintended discrepancies by medication class and route of medication administration, by potential ADE severity, and by proximal cause leading to the discrepancy. The study also assessed potential determinants of unintended discrepancies.

## Methods

This is a pilot prospective study conducted in two tertiary care teaching hospitals in Beirut, Lebanon (Saint George Hospital-University Medical Center and Makassed General Hospital) from March through June 2018.

### Participant selection

Participants were included in the study if they were inpatients aged ≥18 years old, admitted to the Internal Medicine (IM) services, and had ≥1 outpatient medications. Eligible patients were identified on day one of hospital admission through a list generated daily by the Pharmacy Department at each participating hospital. Participants were excluded if they were unresponsive i.e. comatose or patients who could not be interviewed in English or Arabic.

### Data collection process

Third- and fourth-professional-year student pharmacists at the Lebanese American University School of Pharmacy were properly trained to assist in the process. The student pharmacist obtained written consent from eligible patients, and performed face-to-face interviews with patients and/or family members who were willing to participate. Proxy interviews were identified when the patient was not capable of answering questions. In addition to the interview, the student pharmacist was trained to use multiple sources to obtain the BPMH including (but not limited to) examination of home medication bottles or boxes, communication with the treating physician, or review of the patients’ previous medical record, during a previous hospital admission, as applicable. Dietary supplements including vitamins, minerals, and herbal products were also assessed. The information obtained about patients’ medical and medication history was documented on the patient medication reconciliation assessment form (Additional file 1). Subsequently, the student pharmacist checked the medication list in the patient’s medical chart, and compared it to medications he/she had already collected using the assessment form. Upon completion of the data collection, the student pharmacist relayed all findings to the pharmacist at the corresponding site. The on-site pharmacist and/or supervising faculty verified all students’ findings.

The information included in the medication reconciliation assessment form and the tips for conducting the patient medication interview were obtained from the Medications at Transitions and Clinical Handoffs (MATCH) Toolkit for Medication Reconciliation. The MATCH Toolkit for Medication Reconciliation is a public document developed through the support of the Agency for Healthcare Research and Quality (AHRQ), in collaboration with the Joint Commission. This toolkit promotes a detailed approach to medication management and reconciliation that emphasizes standardization of the process for doctors, nurses, and pharmacists within the facility. It documents and confirms a patient’s home medication list upon admission [12]. The Institute for Healthcare Improvement (IHI) recommends the MATCH Toolkit – among other tools – as a guiding material for developing a medication reconciliation process in the hospital or outpatient practice setting [6].

### Main outcomes and measures

The pharmacist identified, analyzed, and classified discrepancies according to the MATCH Toolkit critical thinking process: no discrepancies (one-to-one match), intended discrepancies (discrepancies were appropriate based on the patient’s plan of care) and unintended discrepancies (discrepancies required clarification because there was no explanation based on the patient’s clinical condition or care plan) [12].

In order to limit the bias during categorization of the discrepancies, the pharmacist relied on multiple sources including the BPMH collected by student pharmacists, a review of current and past medical records, and open communication with the interprofessional team to clarify ambiguities when needed. When all home medications, as per the BPMH, were listed on the patients’ medication list, this was deemed to be a one-to-one match with no discrepancies. If the discrepancy was warranted by the clinical condition and plan of care, as documented in the chart, this was deemed to be an intended discrepancy. In case the discrepancy was not justified by the patients’ clinical condition or plan of care, this was deemed to be an unintended discrepancy.

Interventions related to unintended discrepancies were shared with the interprofessional team caring for the patient, including nursing, medical staff and students. The pharmacist communicated directly with the most senior member on the interprofessional team, either an attending physician or senior resident, as available. The pharmacist documented whether the intervention was accepted, rejected or pending review.

The unintended discrepancies were classified by type, medication category, therapeutic/pharmacological class, route of medication involved, and whether or not the discrepancy relates to a high-alert medication as per the Institute of Safe Medication Practices (ISMP) [29]. In addition, the pharmacist estimated the proximal cause leading to the medication discrepancy. In the context of this study, the proximal cause was defined as the apparent reason, or the cause closest in time or sequence to the medication discrepancy, estimated to be the immediate cause of the discrepancy. In contrast to identifying the root causes which usually required conducting structured, robust auditing and feedback method, the proximal cause was estimated by a quick assessment of the pharmacist after review of the unintended discrepancy and soliciting the feedback of the frontline personnel involved the error, during relaying the intervention [30, 31].

Three of the investigators reviewed all unintended discrepancies and came to a consensus to classify each one according to its potential severity. Severity definitions were adapted from a previous study [18]. As such, discrepancies were classified into one of four categories: clinically insignificant (error that would not likely cause harm); clinically significant (error that has the potential to cause harm, and may require increased monitoring); serious (error that has potential to cause harm and 1) likely to require additional intervention or 2) could result in prolonged hospital length of stay); and life-threatening (error having the potential to cause death or likely lead to death without the use of life-sustaining interventions). In case of disagreement, the investigators adopted the least severe classification based on their conservative clinical judgement, in order to avoid overestimation of the study findings.

### Data management and statistical analysis

The data collected was coded, entered into SPSS version 25 software, verified for data entry errors, and analyzed. Descriptive statistics were used to report all participants’ responses.

The dependent variable was the incidence of unintended discrepancies. For the bivariate and multivariable analysis, the “total number of unintended discrepancies” was dichotomized into yes (≥ 1 unintended discrepancy) or no (0 unintended discrepancies). Independent variables tested for their association with the dependent variable consisted of the following: gender, age, creatinine clearance, number of home medications, allergies, previous Adverse Drug Reactions (ADRs), and number of information sources used to obtain the BPMH. The association between categorical variables were evaluated using the Pearson χ2 test or Fisher’s exact test where the expected cell count < 5. Binary logistic regressions were performed using a Backward LR method. Variables with a *p*-value of 0.2 or less in the bivariate analysis were included in the initial models. Results were assumed to be significant when p was < 0.05 for all statistical analysis.

## Results

The study included 204 patients. The patients had equal gender distribution and a mean age of 70.5 years. The majority of the study population (82.4%) took ≥5 home medications. Approximately 16% of patients reported having a history of allergy to ≥1 medication, and around 8% reported experiencing a previous adverse drug reaction. Table 1 details the sociodemographic and baseline characteristics of the patient population.

**Table 1:** Sociodemographic and baseline characteristics

Characteristic	Frequency (Percentage)
Hospital	
Makassed General Hospital	102 (50.0)
Saint George Hospital – University Medical Center	102 (50.0)
Gender	
Male	103 (50.5)
Female	101 (49.5)
Age (years)	
Mean	70.5
Standard deviation	15
Creatinine Clearance (The Cockcroft-*Gault* Equation) [32]	
< 15 mL/min	7 (3.4)
15 mL/min – < 30 mL/min	17 (8.3)
30 mL/min – **<** 50 mL/min	36 (17.6)
≥ 50 mL/min	91 (44.6)
Missing (due to missing serum creatinine, weight, height)	53 (26.0)
Number of Home Medications	
1	6 (2.9)
2	8 (3.9)
3	6 (2.9)
4	16 (7.8)
≥ 5	168 (82.4)
Allergies	
No known drug allergy	172 (84.3)
Yes (to one or more medication)	32 (15.7)
Previous ADRs	
No	163 (79.9)
Yes	17 (8.3)
Don’t Know	24 (11.8)

The time needed to obtain medication history ranged from 3 to 35 min. The student pharmacist used a combination of different information sources to complete the BPMH for each patient: patient interview (70.1%), caregiver/family member interview (57.4%), examination of home medication bottles or boxes (55.9%), review of previous medical record (25.5%), and communication with the physician (3.9%).

Following the critical analysis of patient cases, 94 patient cases (46.1%) had no discrepancies with a complete one-to-one match, and 7.8% included intended discrepancies explained by the patient’s clinical condition. There was 195 unintended medication discrepancies identified in 94 patients by the pharmacy team, with a maximum number of 7 discrepancies per patient case (Table 2).

**Table 2:** Medication reconciliation process

Variable	Frequency (Percentage)
Time Needed For Medication History (min)	
Mean	11.2
Standard deviation	6.2
Information Source^a^	
Patient interview	143 (70.1)
Caregiver / family member interview	117 (57.4)
Examination of home medication bottles or boxes	114 (55.9)
Review of previous medical record	52 (25.5)
Communication with the physician	8 (3.9)
Critical Analysis of Patient Cases	
Cases with no discrepancies (one-to-one match)	94 (46.1)
Cases with intended discrepancies	16 (7.8)
Cases with unintended discrepancies	94 (46.1)
Total number of patient cases	204 (100)

^a^Cumulative percentage exceeds 100%. More than one source of information was used for every patient

Most of the unintended discrepancies (67.7%) involved a prescription medication, and 32.3% involved an over-the-counter medication. The most common discrepancies consisted of medication omission (71.8%), wrong dose (12.8%), wrong medication (7.2%), and wrong frequency (5.1%).

The proximal causes leading to the discrepancies included the patient’s forgetfulness or lack of knowledge (57.9%), the clinician’s lack of knowledge or familiarity with the involved medication (10.3%), a confusion regarding the dosage form of the involved medication (0.5%), and name similarity of the involved medication with another medication (0.5%). In 30.8% of the cases, the hospital pharmacist did not identify a reason behind the discrepancy.

When classifying the unintended discrepancies by route of administration, 92.3% were found to involve an oral medication. Only 3.6% of the unintended discrepancies involved an inhaled medication and 1.5% involved a subcutaneous medication.

The most common agents involved in unintended discrepancies consisted of dietary supplements (27.7%). Other medication classes involved were anti-hyperlipidemic agents (7.2%), medications for reflux disease (7.2%), and medications for asthma/COPD (7.2%). Further details are included in Table 3**.**

**Table 3:** Unintended discrepancies

Variable	Frequency (Percentage) *N* = 195
Unintended Discrepancies by Type	
Omission	140 (71.8)
Wrong dose	25 (12.8)
Wrong medication	14 (7.2)
Wrong frequency	10 (5.1)
Duplication	4 (2.1)
Wrong route	2 (1.0)
Unintended Discrepancies by Medication Route of Administration	
Oral	180 (92.3)
Inhaled	7 (3.6)
Subcutaneous	3 (1.5)
Topical	2 (1.0)
Intravenous	2 (1.0)
Ophthalmic	1 (0.5)
Unintended Discrepancies by Class	
1. Dietary supplements (vitamins, minerals, herbal supplements)	54 (27.7)
2. Medications (prescription and OTC^a^ medications)	
a. Anti-hyperlipidemic agents	14 (7.2)
b. Medications for reflux disease	14 (7.2)
c. Medications for asthma/COPD	14 (7.2)
d. Beta-blockers	10 (5.1)
e. Antidepressants/Anxiolytics	10 (5.1)
f. Oral antidiabetic/Insulin	10 (5.1)
g. Hormonal therapy	9 (4.6)
h. Analgesics	9 (4.6)
i. Medications acting on renin-angiotensin system	6 (3.0)
j. Antibiotics	4 (2.0)
k.Thyroid replacement/Anti-thyroid	4 (2.0)
l. Osteoporosis	4 (2.0)
m. Antiepileptic agents	3 (1.5)
n. Other medications (Anti-diarrheal/Laxatives/Antispasmodics; Anti-arrhythmic agents, anti-gout agents, Aspirin, Antianginal, Corticosteroids, Immunosuppressant, H-1 Blockers, Anticoagulant, Ophthalmic lubricant)	30 (15.3)
Proximal Cause of Unintended Discrepancy	
Patient forgetfulness/Lack of knowledge	113 (57.9)
Clinician lack of knowledge/familiarity with medication	20 (10.3)
Dosage form confusion	1 (0.5)
Name similarity	1 (0.5)
Reason unidentified	60 (30.8)
Potential Severity of Unintended Discrepancy	
Clinically insignificant	122 (62.6)
Clinically significant	71 (36.4)
Serious	2 (1.0)

^a^*OTC* Over-the-counter medication

Moreover, around 8% of the unintended discrepancies involved a high-alert medication, including oral hypoglycemic agents, insulin, anti-arrhythmic agents, and anticoagulants. When assessing potential severity of all medication-related discrepancies by the investigators, 122 discrepancies out of 195 were judged as clinically insignificant, 71 (36.4%) were judged as clinically significant, and only 2 (1%) were judged to be serious. No life-threatening interventions were identified.

Based on the unintended discrepancies found, the pharmacy team recommended a total of 195 medication-related interventions. Amongst these interventions, 64.6% were accepted, 25.6% were rejected, and around 10% pending review. The most common types of medication-related interventions included: the addition of a medication (71.8%), dose adjustment (12.8%), discontinuation or switching of a medication (7.2%), and frequency adjustment (5.1%). Other interventions were giving medication from home supply (1.5%), and adjustment of medication route (1%).

In the multivariable analysis, the number of home medications was the only variable significantly associated with the occurrence of unintended discrepancies (ORa 1.11 (1.03–1.19); *p* = 0.007) (Table 4).

**Table 4:** Unintended discrepancies – multivariable analysis

Variable	Unadjusted OR	ORa	Confidence Interval	*P*-value
Number of information sources	0.464	1.37	0.91–1.70	0.08
Number of home medications	0.102	1.11	1.03–1.19	0.007

Variables with a *p*-value of 0.2 or less in the bivariate analysis were included in the initial model. Those include: Age, Number of information sources used, and Number of home medications

Using a Backward LR method, the model finally retained the variables shown in this table. Hosmer and Lemshow test for sample adequacy *p*-value: 0.882

## Discussion

In this pilot study, the clinical impact of pharmacy-led medication reconciliation performed upon admission to the IM services is examined at two hospitals in Lebanon. During the study period, 204 patients were included, and 195 unintended discrepancies were identified in 94 patients.

The most intricate part of medication reconciliation is obtaining the BPMH. In this study, the student pharmacist used different information sources to complete the BPMH for each patient and mostly relied on patient interviews, caregiver/family member interviews, and examination of home medication boxes. Consulting at least one information source other than the patient is crucial to determine the accuracy of a patient’s medication history list [11]. Other sources that could have been used include outpatient pharmacies, and prescriber offices [16, 33]. In Lebanon, these sources may not provide excellent information to improve BPMH due to the practice of medicine in the country where there is a tendency for patients to seek care directly from specialists (often multiple) without the oversight of a primary-care provider [34]. Furthermore, many patients might not have loyalty to a single community pharmacy and to date, a prescription drug monitoring program database does not exist in Lebanon [35]. The number of information sources remained in the final model of the multivariable analysis for unintended discrepancies (ORa 1.37, *p* = 0.08). The lack of statistical significance may be attributed to the small sample size.

The majority of patients included in this study were polymedicated. In the multivariable analysis, the number of home medications was significantly associated with the identification of unintended discrepancies (ORa 1.11; *p* = 0.007). Based on previous studies, the number of pre-admission medications correlates with admission order errors and unintentional discrepancies [33].

Most of the unintended discrepancies in this study consisted of drug omissions (72%) as similarly reported in the literature [16, 18, 36–39]. In such cases, the patient is failing to receive a pre-admission home medication that was deemed necessary by a HCP. The clinical significance of this omission depends on the drug omitted. The clinical significance of the unintended discrepancies was evaluated on a case-by-case basis. On another note, discrepancies in medication history, such as the omission of medications or incomplete information about adherence or non-adherence, may delay or prevent clinicians from timely identification of drug-induced diagnoses that may have led to the current hospital admission [16].

When assessing potential severity, most of the unintended discrepancies (63%) were judged as clinically insignificant, where the error was less likely to cause harm, such as the omission of a dietary supplement or the omission of an antacid [40]. Around 36% of unintended discrepancies were judged as clinically significant, where the error had the potential to cause harm and may have required additional monitoring, such as omission/wrong dose of a beta-blocker. Discrepancies involving high alert medications such as the omission of an oral hypoglycemic agent were also considered as significant [29]. Two serious discrepancies found in this study involved antiarrhythmic and antithrombotic therapy. The first case involved the omission of amiodarone. The potential severity was classified as serious since antiarrhythmic therapy is useful in reducing the frequency and shortening the duration of arrhythmias, and decreasing arrhythmia-related hospitalizations [41]. Although amiodarone has a long half-life, its omission may be serious as it puts the patient at an increased risk of developing another arrhythmia, or worsening of the original arrhythmia. This also requires additional monitoring including laboratory or diagnostic tests, which may increase length of hospital stay and associated costs. The second serious unintended discrepancy involved the detection of a wrong dose of apixaban, a new oral anticoagulant (NOAC). This severity classification was based on a review of previous studies which showed that off-label dosing of NOACs was associated with morbidity and mortality, and underdosing NOACs was associated with increased cardiovascular hospitalization, and higher stroke rates [42, 43].

Most of the medication-related recommendations relayed by the pharmacy team regarding the unintended discrepancies were accepted (64.6%), and resulted in an alteration in the patient’s pharmaceutical care plan. This finding further supported the role of pharmacists and student pharmacists in the medication reconciliation process. Most of the rejected recommendations were related to the omission of a dietary supplement, which were assessed by the physicians as unnecessary during acute illness and hospitalization.

Furthermore, the investigators estimated the proximal cause of each unintended discrepancy identified. Evaluation of proximal causes helps identify areas of weakness in the medication reconciliation process, where special attention is paid to prevent future discrepancies [19]. Patient forgetfulness or lack of knowledge was the most common cause (57.9%), followed by clinician lack of knowledge or familiarity with the medication (10.3%). The lack of expertise of non-pharmacists in obtaining a proper medication history, and patients who are poor historians are reported as common barriers for medication reconciliation [44, 45]. In this study, there was no identified reason for the medication discrepancy in 30% of the cases. The latter may be attributed to the time-consuming nature of the BMPH process, the lack of collaboration between the different stakeholders, and the limited access to technology and health information systems [44, 45]. In fact, electronic-based systems were shown to improve medication reconciliation processes by facilitating documentation and access to information [46]. Lebanon is an upper middle-income country [47], where the implementation of electronic-based health information systems is lagging behind, across the healthcare sector.

Moreover, in 2019, the Ministry of Public Health in Lebanon in collaboration with the Haute Authorité de Santé (HAS France) updated the Accreditation Standards for Hospitals in Lebanon and emphasized the need for medication reconciliation at admission, and discharge. The medication management chapter within the standards also reinforces that this reconciled list should be shared with the healthcare providers and the pharmacy [48]. Owing to their distinct knowledge and skills as medication experts, pharmacists are uniquely qualified to lead interprofessional efforts, and establish and maintain an effective medication reconciliation process in hospitals [49]. An interprofessional medication reconciliation process revolves around communication, teamwork, and involves the different healthcare professionals in the medication reconciliation process to ensure optimal patient outcomes [50]. For example, pharmacists taking BPMH should not work in silos and need to communicate with the treating physicians to clarify medication orders, and relay any interventions. Moreover, pharmacists are expected to reach out to the patients’ nurses to verify monitoring parameters.

To our knowledge, this is the first published data on medication reconciliation in Lebanon. The study further highlights the importance and the clinical significance of the pharmacist’s role in medication reconciliation and supports the involvement of student pharmacists in medication history taking. The integration of student pharmacists in the collection of patient medication history allows pharmacists some free time to focus on other in-depth clinical activities.

In context of the updated mandatory hospital accreditation standards, the findings of this pilot study were reported to the individual hospital sites, to further support the integration of the pharmacy team in the medication reconciliation process. In collaboration with the study sites, the authors are further planning to assess patient outcomes on transitions of care when comparing pharmacist-driven medication reconciliation versus standard of care medication reconciliation (completed by nurse, physician etc.) in each site. In today’s challenging and fast-paced healthcare environment, healthcare institutions are urged to optimize resources and eliminate duplication of efforts from the patient care process [51]. Although outside the scope of this study, a future research focusing on the cost effectiveness of pharmacist-led medication reconciliation, with or without student pharmacists, in comparison to other health care professionals, may also bring insight to hospital administrators on to how to allocate limited resources.

### Limitations

This study presents several limitations. This was a single arm pilot study where a formal power analysis and a proper control group could not be performed, mainly due to lack of a structured medication reconciliation process in study sites.

The investigators used their clinical judgment to evaluate the proximal causes of discrepancies and to assess their potential severity, which could induce a risk of bias. The investigators recommended interventions solely related to the medication reconciliation process, where renal dosage adjustment was not necessarily assessed. A few of those interventions (9.7%) were not followed upon during the study period, and were reported as pending.

## Conclusions

Unintended medication discrepancies were common on hospital admission to the internal medicine services. Pharmacy-led medication reconciliation upon admission, with student pharmacist involvement and physician communication helped reduce unintended discrepancies and improve medication safety and patient outcomes. Further studies are warranted to assess the impact of pharmacy-led medication reconciliation across transitions of care.

## Additional file

Additional file 1: Patient Medication Reconciliation Assessment Form. The information obtained about patients’ medical and medication history, the medication order form from patient chart, the critical analysis of the discrepancies, and the corresponding interventions. (DOC 283 kb)

## Data Availability

The datasets used and/or analyzed during the current study are available from the corresponding author on reasonable request.

## Abbreviations

ADEs: Adverse drug events
BPMH: Best possible medication history
HCPs: Healthcare professionals
IM: Internal Medicine
IRB: Institutional Review Board
ISMP: Institute of Safe Medication Practices
NOAC: New oral anticoagulant
