# Supplementary material for: Impact of pharmacy-led medication reconciliation on admission to internal medicine service: experience in two tertiary care teaching hospitals
Source: BMC Health Serv Res. 2019 Jul 16;19:493. doi: 10.1186/s12913-019-4323-7 (PMC6636006; doi:10.1186/s12913-019-4323-7)
Supplement: Supplementary file 1 — Patient Medication Reconciliation Assessment Form. The information obtained about patients’ medical and medication history, the medication order form from patient chart, the critical analysis of the discrepancies, and the corresponding interventions. (DOC 283 kb) [file 12913_2019_4323_MOESM1_ESM.doc]

**Additional File 1**

**Patient Medication Reconciliation Assessment Form**

**Patient Demographics:**

**Identification #: ___ Initials:___ Age:___ Gender:___ Wt:___ Ht:___**

**Allergies: SrCr:___ CrCl:___ Information Source: Patient Interview Family Member Previous ADR:________ Number of home meds:___ Previous Medical Record Home Meds Bottles Or Boxes**

**Time needed to collect the medication history: ___ min Other: Specify**

| **Home meds (Dose / Route /**  **Frequency)**  **( including OTC Products and**  **Herbs)** | **Last**  **dose**  **taken** | **Pt has**  **own**  **supply** | **Should**  **continue**  **on**  **admission** | **Was it**  **cont’d** | **Was it**  **D/C** | **Was it**  **substituted** | **Reason for**  **withhold** | **Reason**  **for**  **withhold**  **verified** |
| --- | --- | --- | --- | --- | --- | --- | --- | --- |
|  |  |  |  |  |  |  |  |  |
|  |  |  |  |  |  |  |  |  |
|  |  |  |  |  |  |  |  |  |
|  |  |  |  |  |  |  |  |  |
|  |  |  |  |  |  |  |  |  |
|  |  |  |  |  |  |  |  |  |
|  |  |  |  |  |  |  |  |  |
|  |  |  |  |  |  |  |  |  |
|  |  |  |  |  |  |  |  |  |
|  |  |  |  |  |  |  |  |  |
|  |  |  |  |  |  |  |  |  |
|  |  |  |  |  |  |  |  |  |

Medication order form from patient medical chart:

| **Indication** | **Admission**  **prescriptions** | **Drug/dose/frequency/route** |
| --- | --- | --- |
|  |  |  |
|  |  |  |
|  |  |  |
|  |  |  |
|  |  |  |
|  |  |  |
|  |  |  |
|  |  |  |
|  |  |  |
|  |  |  |
|  |  |  |
|  |  |  |
|  |  |  |
|  |  |  |
|  |  |  |
|  |  |  |

**Critical Analysis of Discrepancies**


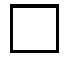
 None. One-to-one Match
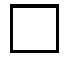
Intended Discrepancies
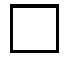
Unintended Discrepancies

**Total Number of Unintended Discrepancies: ­­­­­­­­­­­­­­­­­­­­­­­­­­­­­ ­­­____________**

**Unintended Discrepancies by Medication Category:**


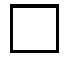
 Over the Counter (OTC)
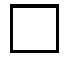
Presciption
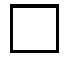
Herbal
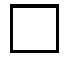
 Allergies

**Unintended Discrepancies by Discrepancy Type:**


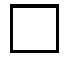
Omission
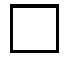
Wrong drug
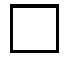
Wrong dose
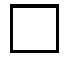
Wrong route
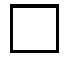
Wrong frequency


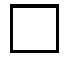
Duplication
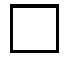
Drug interaction
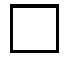
Allergy alert
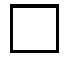
Wrong allergy

**Unintended Discrepancies by Route of Medications involved:**


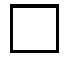
Oral
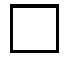
 SC
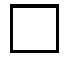
 Buccal
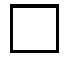
Inhaled
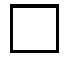
Ophthalmic


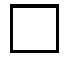
 IM
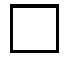
 IV
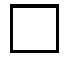
Rectal
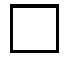
 Otic
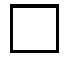
Topical

**Unintended Discrepancies by Therapeutic/Pharmacological Class of Medications involved:**


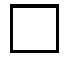
Diuretic
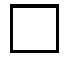
 Lipid Modifying
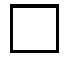
Antipsychotic
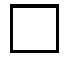
Drug for acid disorders
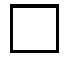
Antibiotic


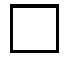
Beta Blocker
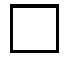
Analgesic
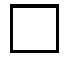
 Oral Antidiabetic
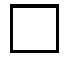
 Vitamin
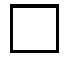
 Mineral Supplements


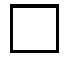
Antiepileptic
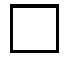
Insulin
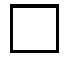
 Antidepressants
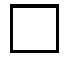
Antidiarrheals/Laxatives/Antispasmodics


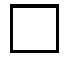
Anti-Parkinson
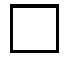
 ACE/ARB
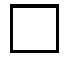
Drugs for asthma/COPD

Other. Specify: ______________________

**Unintended Discrepancy Involving High-Alert Medication**
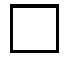
Yes
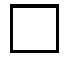
No

**Proximal cause leading to the medication discrepancy** (as determined by the pharmacist)
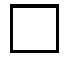
Clinician knowledge (ie, lacking familiarity with medication regimens)


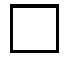
Patient knowledge (ie, lacking familiarity regarding their medication regimens)


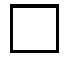
Dosage form confusion


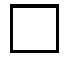
 Brand name and generic name similarity (Look-Alike and Sound-Alike)


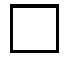
 Unknown (ie, pharmacist unable to determine potential cause leading to medication error)

**Potential severity of the error:**


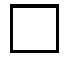
 Clinically insignificant (Error that would not likely cause harm)

Ex: Omission of oral ferrous sulfate, omission of statin, wrong PPI Dose


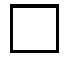
 Significant (have the potential to cause harm and require increased monitoring)

Ex: Wrong dose of antihypertensive


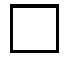
 Serious (have the potential to cause harm and likely to require 1)intervention or 2)prolonged hospital stay)

Ex: Wrong dose of warfarin, omission of levertiracetam for seizure disorder


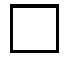
Life-threatening (have the potential to cause death)

Ex: Wrong controlled release morphine death, Nifedipine 90mg immediate release ordered instead of SR.

**Medication-related Interventions:**

Type of intervention:


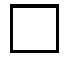
Adjust dose
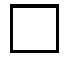
Add a drug
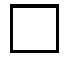
D/C a drug
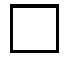
Adjust frequency
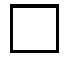
Adjust route


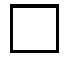
Educate the patient
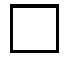
Highlight a drug interactions
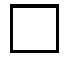
Highlight a drug-disease interactions


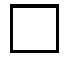
Monitor drug parameter(s)
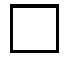
Discontinue drug/give from home supply

Total number of interventions: **_______________________________**


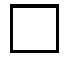
Accepted
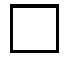
Rejected
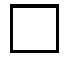
Pending review

| Intervention type | Intervention | Accepted |
| --- | --- | --- |
|  |  |  |
|  |  |  |
|  |  |  |
|  |  |  |
